# Soil Microbial Community Structure and Carbon Stocks Following Fertilization with Organic Fertilizers and Biological Inputs

**DOI:** 10.3390/biology13070534

**Published:** 2024-07-17

**Authors:** Diana Sivojienė, Aistė Masevičienė, Lina Žičkienė, Almantas Ražukas, Audrius Kačergius

**Affiliations:** Lithuanian Research Centre for Agriculture and Forestry, Kėdainiai District, LT-58344 Akademija, Lithuania; aiste.maseviciene@lammc.lt (A.M.); lina.zickiene@lammc.lt (L.Ž.); almantas.razukas@lammc.lt (A.R.); audrius.kacergius@lammc.lt (A.K.)

**Keywords:** organic fertilizers, soil microbiome, metagenomic sequencing, diversity of soil microbial population

## Abstract

**Simple Summary:**

Soil and its quality are becoming more and more important. Healthy and fertile soil allows for the cultivation of high-quality food plants, is resistant to erosion, and contributes to the mitigation of climate change. Researchers are intensively working on research that would allow not only to maintain the quality of the soil, but also to achieve the highest possible yield. Recently, a number of studies have been conducted to determine the benefits of organic fertilizers on soil properties and yield. Our research is about how organic fertilizers and biological inputs affect one of the most important soil components, soil bacteria and fungi, and how other agrochemical parameters change after the application of organic fertilizers. We present a lot of information on how the values of the studied agrochemical and microbiological indicators changed, depending on the use of different organic fertilizers. We also talk about the established effects of the studied biological inputs. Our results contribute to the body of knowledge and can be used to implement sustainable agriculture.

**Abstract:**

The application of organic fertilizers and biological inputs to soil inevitably affects its quality, agrochemical indicators, and microbiota. Sustainable agriculture is based on continuously learning about how to properly manage available soil, water, and biological resources. The aim of the study was to determine changes in microorganism communities and carbon stocks in infertile soils for fertilization using different organic fertilizers and their combinations with bio-inputs. Genetic analysis of microorganism populations was performed using the NGS approach. Our study showed that the application of organic fertilizers affects the soil microbiota and the taxonomic structure of its communities. Specific groups of bacteria, such as *Bacillota*, were promoted by organic fertilization, meanwhile the abundance of *Pseudomonadota* and *Ascomycota* decreased in most treatments after the application of poultry manure. Metagenomic analysis confirmed that the use of bio-inputs increased the relative abundance of *Trichoderma* spp. fungi; meanwhile, a significant change was not found in the representatives of *Azotobacter* compared to the treatments where the bio-inputs were not used. The positive influence of fertilization appeared on all the studied agrochemical indicators. Higher concentrations of C_org_ and N_min_ accumulated in the soil when we used granulated poultry manure, and pH_KCl_ when we used cattle manure.

## 1. Introduction

Agriculture is a sector that has a significant impact on our environment and is one of the ways to ensure the production of essential food crops [1]. Ecological knowledge and good farming practices can be used as tools to maintain as much as possible natural ecosystems [2]. Good farming practices help protect soil fertility, water resources, and biodiversity, and help increase the amount of produce grown, while reducing widespread land degradation [3]. The growing problem of soil degradation is prompting a new review of the application of organic fertilizers in agriculture. Manure and other organic fertilizers are well known additives for maintaining and improving soil quality [4,5]. The positive impact of organic granulated fertilizers on soil is primarily shown by the increase in the amount of nutrients and organic matter, which is particularly important in intensive agriculture [6]. It also, at the same time, improves the physical, chemical, and biological properties of soil [7,8,9,10]. With organic fertilizers, in addition to organic matter and basic macronutrients (nitrogen, phosphorus, and potassium), the soil is supplemented with calcium and magnesium micronutrients [11,12,13]. This complex addition of food elements comprehensively optimizes plant nutrition and provides nutrients for microbial growth [14,15,16]. All this helps to support natural processes in the soil, which will have long-term effects on soil sustainability and fertility [17], directly affecting plant growth and increasing productivity [9,10,18,19].

The rational use of organic fertilizers is based not only on improving soil properties, but also on reducing the negative impact on the environment and negative climate changes [20,21]. Organic fertilizers are considered long-term sources of nutrients due to their slow decomposition of organic matter and the release of plant-available nutrients [17,22,23,24]. Thus, due to slow mineralization, mineral nutrients, especially mineral nitrogen, from organic fertilizers leach into groundwater more slowly than from mineral fertilizers, because of which water contamination due to nitrogen and other compounds is reduced [25]. New manure granulation technologies are being intensively developed to avoid the potential loss of nutrients in the soil of the previously used fallow manure [8]. This encourages the improvement of the processing of manure with a high moisture content into manure pellets with a high dry matter content, which are easier to transport and incorporate into soil [23,26].

Soil is a habitat to a variety of microorganisms, including bacteria, archaea, and fungi [27]. Bacteria and fungi are critical components of agricultural systems [28]. They play important roles in biogeochemical cycling and are distributed throughout soil [29,30]. Being decomposers, mutualistic or antagonistic symbionts, they influence the growth and health of other organisms [30,31]. Due to climate change and other anthropogenic factors, soil microorganisms are faced with the challenge of adapting to these changes [32].

Soil microorganisms are the main providers of soil functions and consequent services, such as carbon and other nutrient cycling methods. Currently, there is still a limited understanding of the impacts of the temporal and spatial dynamics of soil microorganisms on long-term biogeochemical soil processes, which depend not only on land cover, but also on climate [33]. Microbial metabolism has been shown to be the basis of soil organic matter (SOM), and its composition is the main driver of soil stability. Studies by various researchers have demonstrated the extraordinary importance of soil microorganisms in the creation of persistent long-term SOM fractions [34,35]. Based on the insights from as many as 197 peer-reviewed publications, the net benefits of agricultural management to improve soil carbon sequestration would not be sustainable if the role of soil microbial communities was ignored. The restoration of degraded soil microbial communities in agricultural soils is essential to maintain long-term high C potentials and to stabilize them over time [36].

The challenges posed by climate change are becoming more and more relevant and the importance of sustainable agriculture is increasing. Fertilization with organic fertilizers and the possibility to use suitable biological inputs are mentioned as an opportunity to contribute to sustainable farming. Organic fertilizers are considered long-term sources of nutrients, and mineral nitrogen from these fertilizers leaches into groundwater more slowly; therefore, it is a positive activity in relation to climate change. Since fertilization and the knowledge of the processes occurring in the soil after fertilization can help to ensure long-term positive effects on soil quality and fertility, the aim of this study was to investigate how the applications of organic fertilizers (litter, granulated poultry, and cattle manure) and biological inputs affect soil carbon stocks and the functional and taxonomic compositions of soil bacteria and fungi in an experimental agricultural field.

## 2. Materials and Methods

### 2.1. Study Site and Soil Sampling

Soil samples from the experimental agricultural field, located in southeastern Lithuania (54.609344, 25.125962), were selected for metagenomic analysis and soil agrochemical analysis. According to the international soil classification system, the soil in the investigated area was sandy loam Haplic Luvisol [37]. A total of 22 samples for metagenomic analysis was collected in the summer period (July) of the years 2020 (11 samples) and 2022 (11 samples). Soil samples were taken in four replicates of the relevant test from an arable layer depth of 10–20 cm. A pooled sample of the relevant test was formed from four replicates. For the determination of pHKCl and organic carbon (Corg), the soil samples were taken from a soil depth of 0–20 cm and for the determination of mineral nitrogen (Nmin) from a soil depth of 0–60 cm.

All the mentioned samples were taken from the experimental field, which was fertilized with various organic fertilizers and their combinations with biological inputs or mineral fertilizers. The descriptions of fertilizers that were used in the experimental agricultural field are presented in Table 1. The bio-inputs used in the treatment were as follows: No. 1—*Azotobacter* spp.—a mixture of two cultures: *A. chroococcum* and *A. vinelandii*, together with the residues of the culture medium; No. 2—*Trichoderma* spp.—a mixture of three cultures: *Trichoderma harzianum*, *T. tomentosum*, and *T. viride*. They were selected based on their operational specificity and wide range of applications [38,39,40,41,42,43,44]. Fungal and bacterial strains were previously isolated from soil and prepared specifically for this research.

C_org_ and pH_KCl_ were tested in the fall periods of 2020 and 2022, after harvesting the crops (spring barley and spring wheat, respectively); meanwhile, N_min_ was tested in the spring. Various litter and granular organic fertilizers were applied during the experiment and their combinations with biological inputs and mineral fertilizers were applied to the soil 2 times—at the beginning of this research (2018) and after two years (2020).

### 2.2. Climate Conditions

**Figure 1:**
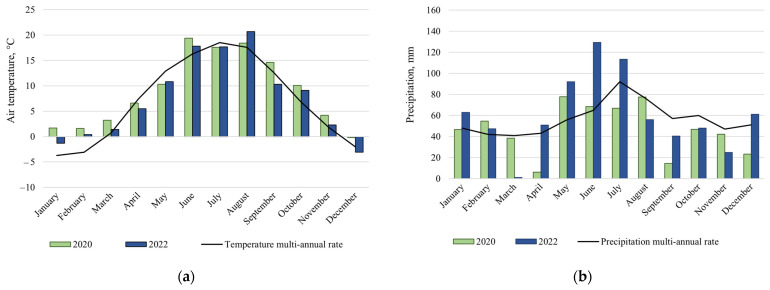
Average temperature (°C) (**a**) and average precipitation (mm) (**b**) in 2020 and 2022.

According to the data of the Lithuanian Hydrometeorological Service [45], the average air temperature in Lithuania in 2020 was 9.2 °C, and in 2022—7.9 °C (1.8 °C and 0.5 °C more than the multi-annual rate—MAR (1990–2020 MAR air temperature was 7.4 °C)) [46]. The average amount of precipitation in 2020 was 646 mm (49 mm below the MAR (695 mm)), and in 2022—674 mm, close to the MAR. Graphic images are shown in Figure 1.

In July 2020, the average air temperature in Lithuania was 17.2 °C (1.1 °C below the MAR). Other summer months of this year were warmer and exceeded the MAR. July in the year 2022 was slightly warmer than in 2020 and was 17.7 °C (0.6 °C below the MAR). As in 2020, other summer months of the year 2022 were warmer and exceeded the MAR. In 2020, 63 mm (0.8 of the MAR) of precipitation fell on average in Lithuania; meanwhile, in 2022, the level was much higher—106.7 mm (1.27 of the MAR). Considering the entire summer period, 2022 was wetter than 2020.

### 2.3. Soil Agrochemical Analysis

The determination of soil pH was performed using a 1:5 (*v*/*v*) soil suspension in 1 M KCl. The mixture was shaken for 60 min and left to sit for 1 h. The pH of the suspension was measured at 20 ± 2 °C by stirring with a pH meter [47]. Organic carbon (C_org_) was determined according to ISO 10694:1995 [48]; dry combustion was determined with total carbon analyzer Liqui TOC II. Mineral nitrogen (Nmin) was extracted in a 1:5 (*w*/*v*) soil suspension of the 1 M KCl solution. The suspension was shaken for 60 min at 20 ± 2 °C. After shaking, the suspension was filtered and analyzed using a flow injection analysis (FIA) system by an FIASTAR5000 analyzer. Nmin was calculated by adding the sum of nitrate and nitrite nitrogen to ammonia nitrogen [49].

### 2.4. Soil DNA Extraction and Microbiomic Analysis

DNA was extracted from homogenized soil samples using a ZymoBIOMICS^®^-96 MagBead DNA Kit (Zymo Research, Irvine, CA, USA) following the manufacturer’s instructions. The DNA samples were prepared for targeted sequencing with a Quick-16S™ Primer Set V3-V4 (Zymo Research, Irvine, CA, USA) and ZymoBIOMICS^®^ Services ITS2 Primer Set (Zymo Research, Irvine, CA, USA). The sequencing library was prepared using a library preparation process, where PCR reactions were performed in real-time PCR machines to control cycles and therefore limit PCR chimera formation. The final PCR products were quantified with qPCR fluorescence readings and pooled together based on equal molarity. The final pooled library was cleaned up with the Select-a-Size DNA Clean & Concentrator™ (Zymo Research, Irvine, CA, USA), then quantified with TapeStation^®^ (Agilent Technologies, Santa Clara, CA, USA) and Qubit^®^ (Thermo Fisher Scientific, Waltham, WA, USA). The final library was sequenced on Illumina^®^ MiSeq™ with a v3 reagent kit (600 cycles).

### 2.5. Bioinformatics Analysis

Unique amplicon sequences were inferred from raw reads using the Dada2 pipeline [50]. Chimeric sequences were also removed with the Dada2 pipeline. Taxonomy assignment was performed using Uclust from Qiime v.1.9.1. Taxonomy was assigned with the Zymo Research Database, a 16S database that is internally designed and curated, as reference. Composition visualization, alpha diversity, and beta diversity analyses were performed with Qiime v.1.9.1 [51]. If applicable, taxonomy that had significant abundance among different groups was identified by LEfSe [52] using default settings. Other analyses, such as heatmaps, Taxa2SV_deomposer, and PCoA plots, were performed with internal scripts.

### 2.6. Statistical Analysis

The research results were evaluated by the method of analysis of variance (ANOVA), applying Duncan’s Multiple Range Test-R Studio. When assessing the statistical reliability of the data, the absence of the same letters between the compared treatments of the experiment indicates that the differences between the mentioned treatments are essential, and, on the contrary, in the case of identical correspondences of the letters, they are insignificant.

Alpha diversity metrics were used to express soil microbial community structure. The Shannon diversity index measures both the number of species and the disparity between species abundance. Alpha diversity analysis was performed with Qiime v.1.9.1 [51].

## 3. Results

### 3.1. Soil Agrochemical Analysis

The main nutrients from various types of organic fertilizers are released into compounds absorbed by plants with different intensities, so it is important to study and evaluate the changes in the agrochemical properties of soil (mineral nitrogen, organic carbon, and soil pH_KCl_) in the case of different fertilizing methods with organic fertilizers and their combinations with biological preparations. The conducted studies showed that fertilizing with various organic fertilizers of poultry or cattle manure and their combinations with biological inputs influenced all studied agrochemical properties of soil–organic carbon (C_org_) and mineral nitrogen (N_min_)—and soil pH_KCl_—partly.

**Figure 2:**
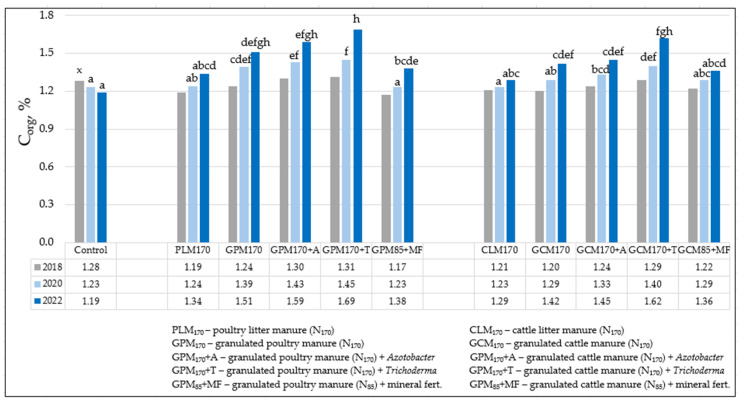
The influence of various organic fertilizers and their combinations with biological inputs on changes in organic carbon (C_org_) in the 0–20 cm soil layer (autumn 2018, 2020, and 2022). Note. Significant differences were identified between experimental data marked a, b, c, d, e, f, g, h at the 5% probability level (*p* ≤ 0.05). When assessing the statistical significance of the data, the absence of the same letters (a–h) between the compared variants of the experiment indicates that the differences between the mentioned variants are significant, and on the contrary, in the case of identical letter correspondences, they are insignificant. _X_ —the results were presented before the installation of the experiment (in 2018), so no statistical evaluation of the data was performed; only the background level of C_org_ in the soil was evaluated.

Changes in organic carbon (C_org_) in the soil were determined by both different organic fertilizers and their combinations with biological preparations (Figure 2). In the control unfertilized fields (Cs), C_org_ tended to decrease by 0.09% per unit during the four-year study period, and, at the end of the experiment (2022), was 1.19%. Meanwhile, when fertilizing with different forms of poultry manure and their combinations with biological inputs, C_org_ in the soil varied from 1.34 to 1.69%, and when fertilizing with various cattle manure and its combinations—1.29–1.62%. This indicator was most effectively increased in the soil by both granulated organic poultry and cattle manure fertilizers (GPM_170_ + T and GCM_170_ + T) in combination with a biological input (T) containing *Trichoderma* spp. fungi. The changes compared to the unfertilized field (C) were 0.50 and 0.43% units, and compared to GPM_170_ and GCM_170_—0.18 and 0.20% units more, respectively. Also, slightly higher concentrations of C_org_ accumulated in the soil when plants were fertilized with the mentioned organic fertilizers, GPM_170_ and GCM_170_, both without and in combination with the biological input (A), which includes nitrogen-fixing bacteria *Azotobacter* spp. Mineral fertilizers applied to the soil in combination with both granulated poultry (GPM_85_) and cattle (GCM_85_) half-rate manure also tended to increase the accumulation of C_org_ in the soil, but not as markedly.

The experimental sites belong to the soil zone of Southeast Lithuania, which is rich in poor C_org_ soils. Evaluating the changes in C_org_ during the research period shows the positive effect of fertilization with organic fertilizers on the accumulation of this indicator in the soil. Soil analysis performed prior to experiment installation showed that most of the sandy loam *Haplic Luvisol* had medium (1.21–1.31%) C_org_ concentrations, with a 0–20 cm soil layer, and only isolated plots had low concentrations (1.17–1.20%). At the end of the experiment, a low concentration of C_org_ in the soil was found only in the control unfertilized fields, while in the fertilized fields it was medium and the range of values in the soil increased and varied from 1.29 to 1.69%. C_org_ accumulated in the arable layer (0–20 cm) after the first application of organic fertilizers in autumn 2018 and tended to increase every year until the end of the experiment. The change during the research period in the fields fertilized with various organic fertilizers varied on average from 0.08 to 0.38% units. During the research period, the biggest changes (0.38 and 0.33% units, respectively) were recorded in the fields, when plants were fertilized with granulated poultry or cattle manure in combination with a biological input (T) containing *Trichoderma* spp. Biological input (A) with nitrogen-fixing bacteria *Azotobacter* spp. also promoted the accumulation of C_org_ in the soil, but not as intensively as with *Trichoderma* spp. Poultry litter (PLM_170_) and cattle litter (CLM_170_) manure fertilizers, as well as mineral fertilizers (MF) incorporated into the soil in combination with both granulated poultry (GPM_85_) and cattle (GCM_85_) half-rate manure, had the least influence on C_org_ changes in the soil during the study period (0.08–0.21% units).

**Figure 3:**
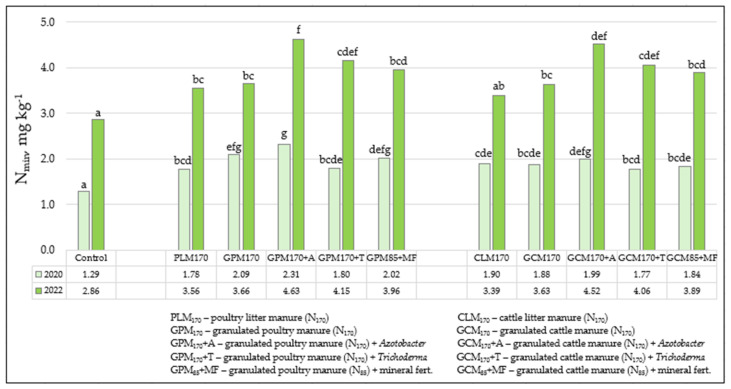
The influence of various organic fertilizers and their combinations with bio-inputs on the concentration of mineral nitrogen (N_min_) in the 0–60 cm soil layer (spring 2020 and 2022). Note. The average N_min_ concentration before the installation of the experiment in the fall of 2018 (before the introduction of various organic fertilizers) was 4.63 mg kg^−1^; the range of values—4.37–4.94 mg kg^−1^. Significant differences were identified between experimental data marked a, b, c, d, e, f, g at the 5% probability level (*p* ≤ 0.05). When assessing the statistical significance of the data, the absence of the same letters (a–g) between the compared variants of the experiment indicates that the differences between the mentioned variants are significant, and on the contrary, in the case of identical letter correspondences, they are insignificant.

Mineral nitrogen (N_min_), especially its nitrate form, which makes up the majority, is extremely mobile in soil, so the concentrations of this element in spring can be influenced by various factors, such as fertilization; soil type and its granulometric composition (texture); cultivated plants; and meteorological conditions, especially rainfall, frost depth during the winter, etc. As we can see in Figure 3, in all years of the experiment, N_min_ concentrations, according to their evaluation scale in the 0–60 cm soil layer, fluctuate between very low and low values. The average concentration of N_min_ before the installation of the experiment in the autumn of 2018 before the application of various organic fertilizers was low and reached 4.63 mg kg^−1^, and the limits of variation in the values varied from very small to small—4.37–4.94 mg kg^−1^. Almost 2 years after both the first and second applications of organic fertilizers to the soil, N_min_ concentrations in 2020 in spring were very low, and in 2022—very low and low. It is likely that part of the released nitrogen was used for the formation of plant crops, the other part could leach into the deeper layers of the soil due to the predominant light granulometric composition (texture) of the soil, and the rest simply remained in the soil in the form of organic matter. However, in the spring of 2022, the concentrations of this mobile element in the soil were on average 2-times higher than in 2020, and this was influenced by the meteorological conditions that prevailed in the late autumn and winter periods (November–March). In 2022, the amount of precipitation during the mentioned period was 31 mm higher than in 2020, but in recent years, no negative air temperatures were recorded, which allowed N_min_ to easily migrate from the upper layers of the soil with a lighter granulometric composition (texture) to deeper ones, and then to groundwater. In 2022, the situation was the opposite, because the negative temperatures that prevailed in December 2021 and January 2022 allowed the formation of frost in the soil, which slowed down the processes of N_min_ leaching from the soil, which is why the reserves were slightly higher in the spring. The processes of intensive washing in the soil were also accelerated by its light granulometric composition, especially the sand on the gravel in the subsoil.

Fertilization with various organic fertilizers in combination with bio-inputs or mineral fertilizers also influenced the changes in N_min_ in the soil (Figure 3). The efficiency of fertilizers was slightly more pronounced in 2022, not only because of the higher concentration of this mobile element in the soil, but also, in comparison with the unfertilized soil, the increase in the fertilized experimental fields was higher than in the ones studied in 2020. Due to the influence of various poultry manure fertilizers and their combinations with biological preparations and mineral fertilizers, in 2022, the increase in N_min_ in fertilized fields was determined to be 1.13 mg kg^−1^ higher on average compared to the unfertilized control field, and in 2020—0.71 mg kg^−1^ higher. When fertilizing with various forms of cattle manure, the increase compared to the control (C) was slightly lower: in 2020—0.59 mg kg^−1^, and in 2022—1.04 mg kg^−1^. Evaluating the effectiveness of different organic fertilizers, in the 0–60 cm layer of soil, more of this plant food material was found when fertilizing plants with both litter and granulated poultry manure fertilizers and in combination with bio-inputs than in fields fertilized with cattle manure. The highest N_min_ concentrations were determined in 2020 after GPM_170_ + A and GPM_170_ were added to the soil, and in 2022 when GPM_170_ + A and GPM_170_ + T were fertilized. When fertilizing plants with granulated cattle manure, it was also the most effective in 2020 in combination with the biological preparation of *Azotobacter* spp. (GCM_170_ + A), and in 2022 by fertilizing GCM_170_ + A and GCM_170_ + T.

Evaluating the effectiveness of biological preparations on the concentration of N_min_ in the soil, almost 2 years later, both after the first and after the second applications of organic fertilizers to the soil, only the biological preparation with nitrogen-fixing bacteria *Azotobacter* spp. (A) trended to increase the concentration of N_min_ in the soil (Figure 3). It worked effectively when used in combination with both GPM_170_ and GCM_170_, as significant differences were found not only when compared to the control box (C), but also with GPM_170_ or GCM_170_. The effect of the bio-input containing *Trichoderma* spp. (T) was less regular and pronounced, and in 2020, when it was used in combination with both GPM_170_ and GCM_170_ N_min_, it was further reduced in the soil than when fertilizing only with granulated poultry or cattle manure at a 170 kg ha^−1^ norm. The effectiveness of the introduction of the bio-input with *Trichoderma* spp. into the soil became evident only at the end of the experiment (in 2022).

**Figure 4:**
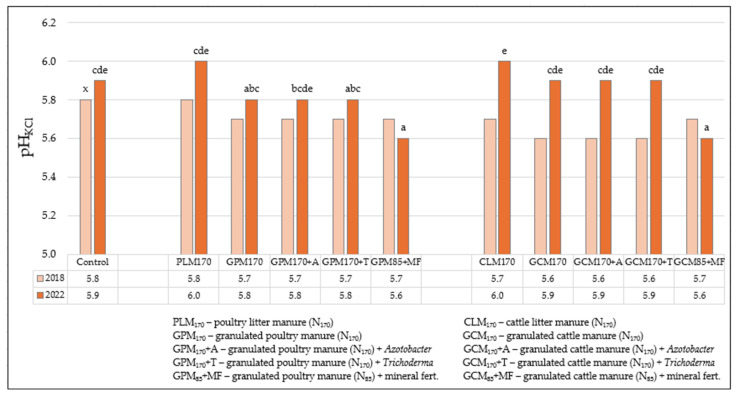
The influence of various organic fertilizers and their combinations with bio-inputs on pH_KCl_ changes in the 0–20 cm soil layer (autumn 2018 and 2022). Note. Significant differences were identified between experimental data marked a, b, c, d, e at the 5% probability level (*p* ≤ 0.05). When assessing the statistical significance of the data, the absence of the same letters (a–e) between the compared variants of the experiment indicates that the differences between the mentioned variants are significant, and on the contrary, in the case of identical letter correspondences, they are insignificant. x—chemical analyses of the soil were carried out before the installation of the experiment, i.e., before the introduction of organic fertilizers into the soil; therefore, the statistical evaluation of the data in 2018 was not performed as only the background was assessed.

When evaluating the effectiveness of various organic fertilizers and their combinations with bio-inputs on soil pH_KCl_, it can be seen that the differences between the field options are not very pronounced, but when evaluating the changes in this indicator over time, at the end of the experiment (in 2022), positive trends of increasing pH_KCl_ emerged, except for the fields where plants were fertilized with half rates of granulated poultry (GPM_85_) and/or granulated cattle manure (GCM_85_) in combination with mineral fertilizers (MF) (Figure 4). At the beginning of the experiment, before the application of fertilizers to the soil, the pH_KCl_ of the soil in all fields was moderately acidic and ranged from 5.6 to 5.8, and at the end of the experiment it also remained moderately acidic, but the limits of variation in the values slightly increased and were in the range of 5.6–6.0. Both litter and granulated cattle manure organic fertilizers had a higher effect on pH_KCl_ changes in the soil when they were evaluated during the experiment, and compared to fields fertilized with various poultry manures, this indicator increased by 0.3 over the corresponding 4-year period. The influence of bio-inputs both with *Trichoderma* spp. and with *Azotobacter* spp. on the changes in the mentioned indicator in the soil did not occur. It is also important to pay attention to the fact that soil pH_KCl_ is also determined by soil typology, its granulometric composition (texture), and the depth of carbonate subsidence, which usually subsides at a depth of 0.8–1.2 m in the soils of the Eastern zone of the country.

### 3.2. Soil Microbiomic Analysis

**Figure 5:**
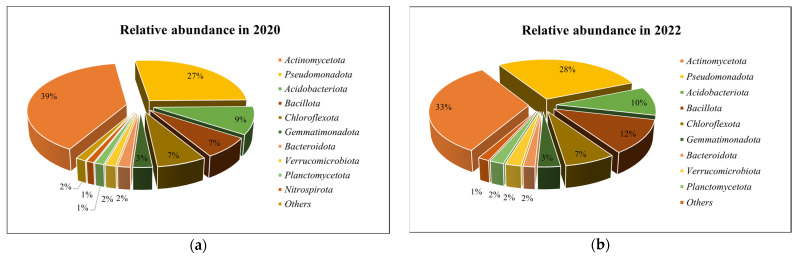
Relative abundances of the most common bacterial phyla in 2020 (**a**) and 2022 (**b**).

Illumina^®^ MiSeq™ analysis of soil samples from the experimental agricultural field (22 samples) revealed 658,451 high-quality sequences (259,900 bacterial sequences in 2020, and much more bacterial sequences in 2022—393,355). All sequences were separated into 2588 OTUs. 2581 (99.7%) OTUs were classified as *Bacteria*. In 2020, the number of bacterial OTUs was 1341, and in 2022 much more—2358 OTUs. Average bacterial OTU richness per sample in 2020 was 438, in 2022—730. Bacterial OTU was assigned to the phylum level. A total of 22 phyla were detected in the bacterial community (in 2020—20 phyla, in 2022—21 phyla). A total of 10 phyla in 2020 and 9 phyla in 2022 can be considered dominant, with a relative abundance of more than 1%. The relative abundances of the most common bacterial OTUs at the phylum level in 2020 and 2022 are presented in Figure 5.

The most abundant bacterial phyla were *Actinomycetota* (39% of bacterial sequences in 2020, and 33% in 2022), *Pseudomonadota* (27%, 28%), *Acidobacteriota* (9%, 10%), *Bacillota* (7%, 12%), and *Chloroflexota* (7%, 7%). *Actinomycetota* dominated at the class level (57.5% of all *Actinomycetota* in 2020 and 54.4% in 2022).

**Figure 6:**
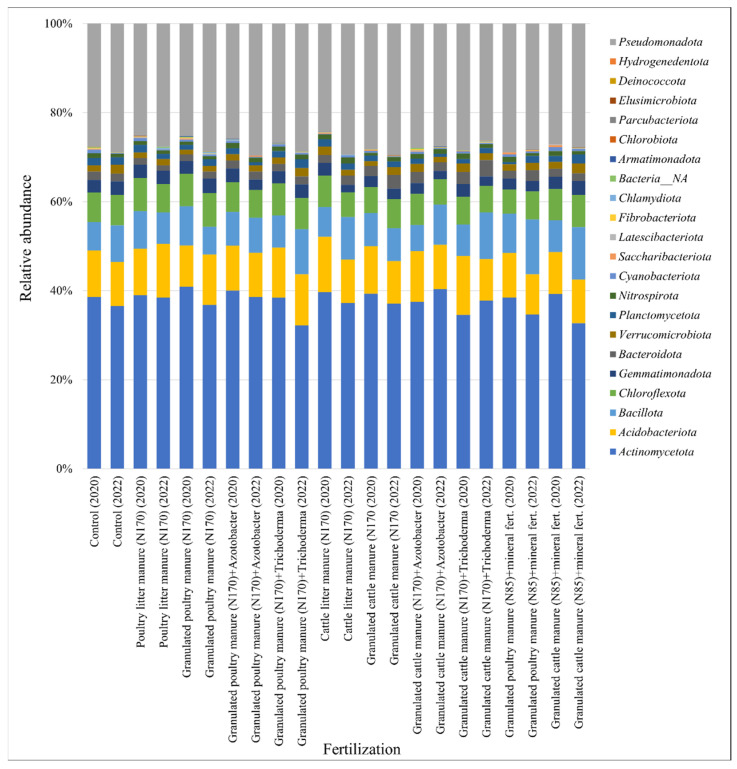
Relative abundances of the most common bacteria phyla in different fertilization options in 2020 and 2022.

Evaluating the abundance of bacteria in different fertilization options, it was found that previously mentioned dominant phyla dominated in all fertilization treatments, but representatives of non-dominant phyla were only present in some fertilization treatments and were not detected in the control field (Figure 6). *Hydrogenedentota* was found only in the GCM_170_ + T fertilized field; *Elusimicrobiota* only in PLM_170_, GPM_170_ + A, GPM_170_ + T, GCM_170_, GPM_85_ + MF, and GCM_85_ + MF; and *Chlamydiota* was found in PLM_170_, GPM_170_, GPM_170_ + A, GPM_170_ + T, CLM_170_, GCM_170_ + T, GPM_85_ + MF, and GCM_85_ + MF.

After applying poultry manure fertilizer to the soil, a decrease in *Actinomycetota* occurred in GPM_170_ + T and GPM_85_ + MF. After applying cattle manure fertilizers, decreases occurred in GCM_170_ + A (in 2020), GCM_170_ + T (in 2020), and GCM_85_ + MF (in 2022). Meanwhile, the abundance of *Pseudomonadota* decreased with most treatments after the application of poultry manure. Only GPM_170_ + A (in 2022) and GPM_85_ + MF (in 2020) remained like the control. In CLM_170_ (in 2022), GCM_170_, GCM_170_ + A (in 2020), and GCM_170_ + T (in 2020), an increase in *Pseudomonadota* was observed. In 2020, *Bacillota* increased in all treatments after fertilizer application (except for one—GCM_170_ + A). In 2022, *Bacillota* also increased following most treatments, except PLM_170_, GPM_170_, and GCM_170_. In the fertilized fields, GPM_170_ + A and GCM_170_ + A, where the bio-input *Azotobacter* was additionally applied, metagenomic analysis did not detect representatives of *Azotobacter* during the taxonomic assignment, although it was expected.

**Figure 7:**
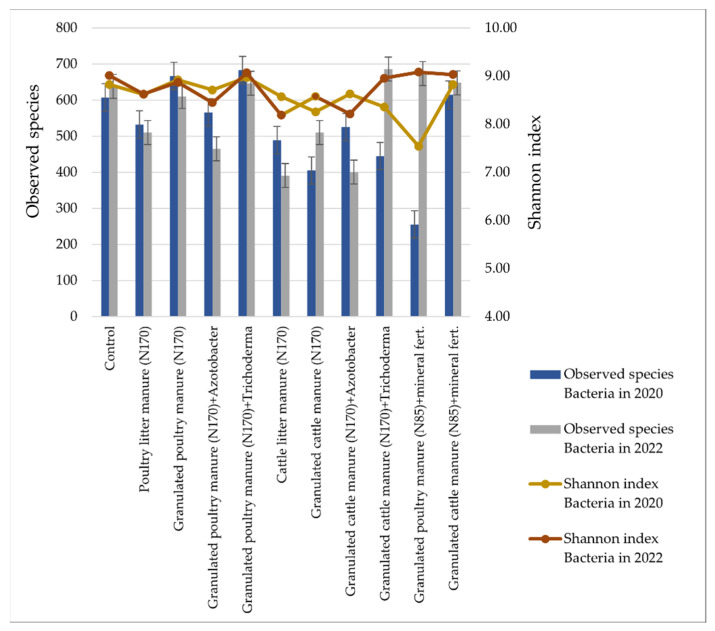
Bacterial alpha diversity parameters (observed species and Shannon index) for the different fertilization options.

The lowest diversity of bacterial species was found in soil samples CLM_170_ and GCM_170_ (Figure 7). The highest diversity of bacterial species was in GPM_170_ and GPM_170_ + T. The Shannon index of species diversity also varied between treatments and was highest in GPM_170_ + T and lowest in GPM_85_ + MF (in 2020).

**Figure 8:**
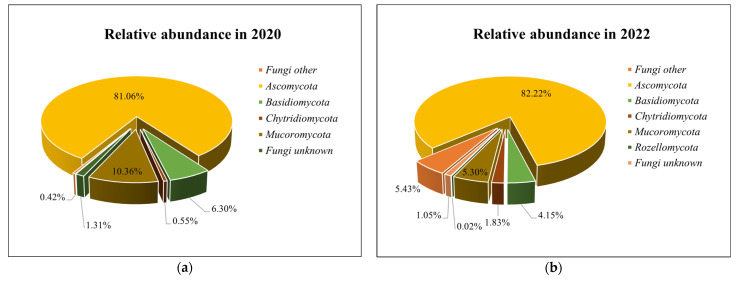
Relative abundances of the most common fungi phyla in 2020 (**a**) and 2022 (**b**).

On average, 97,854 high-quality fungal sequences were obtained from each experimental agricultural field sample. All fungal sequences were separated into 613 OTUs classified as *Fungi*. Average fungal OTU richness per sample in 2020 was 135, in 2022—168. Fungal OTUs were assigned to the phylum level (Figure 8).

The most abundant fungal phyla were *Ascomycota* (81.06% of fungal sequences in 2020 and 82.22% in 2022), *Mucoromycota* (10.36%, 5.30%), and *Basidiomycota* (6.30%, 4.15%). In *Ascomycota*, at the class level, *Sordariomycetes* dominates (53.93% of all *Ascomycota* in 2020 and 60.56% in 2022). It was also observed that *Mucoromycota* in 2020 was almost twice as abundant as in 2022. In 2022, the relative abundance of other fungi was significantly higher than in 2020.

**Figure 9:**
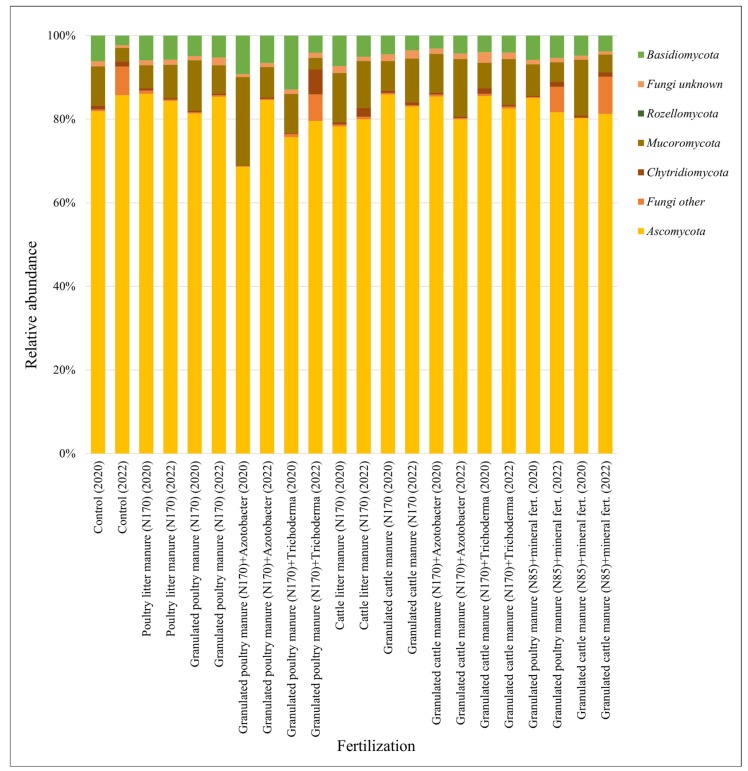
Relative abundances of the most common fungi phyla in different fertilization options in 2020 and 2022.

The results of the different fertilization options show that all found phyla are present in all fertilization treatments, except *Rozellomycota. Rozellomycota* representatives were found only in 2022 in GPM_170_ + T, CLM_170_, GCM_170_ + A, GCM_170_ + A, and GCM_85_ + MF fertilized fields (Figure 9).

*Ascomycota* decreased in most treatments after the application of poultry manure fertilizer; only in PLM_170_ (2020) and GPM_85_ + MF (2020) a slight increase in abundance was observed. In treatments after the application of cattle manure fertilizer, in GCM_170_ (2020), GCM_170_ + A (2020), and GCM_170_ + T (2020), an increase in abundance of *Ascomycota* was observed. A decrease in relative abundance was also observed for all other treatments with cattle manure fertilizers. In 2020, an increase in representatives of *Basidiomycota* was observed in GPM_170_ + A, GPM_170_ + T, and CLM_170_. *Mucoromycota* was more abundant in most treatments after the application of organic fertilizers. Among all fertilization options, GPM_170_ + T stood out; here, both in 2020 and 2022, the abundance of *Mucoromycota* representatives decreased compared to the control.

In the year 2020, at the genus level, 372 OTUs, classified as *Trichoderma*, were identified in the GPM_170_ treatment without *Trichoderma*, and 468 OTUs in the GPM_170_ + T treatment with the bio-input *Trichoderma*. In the year 2022, the GPM_170_ treatment has 281 OTUs, and GPM_170_ + T has 2273 OTUs. Analyzing the GCM_170_ fertilization option without the bio-input *Trichoderma*, it was found that, in 2020, 488 OTUs, classified as *Trichoderma* at the genus level, were detected here. In the GCM_170_ + T fertilization option with bio-input *Trichoderma*, 692 OTUs, classified as *Trichoderma,* were detected. However, in the year 2022, more *Trichoderma* representatives were detected in GCM_170_ (412 OTUs) than in GCM_170_ + T (323 OTUs).

**Figure 10:**
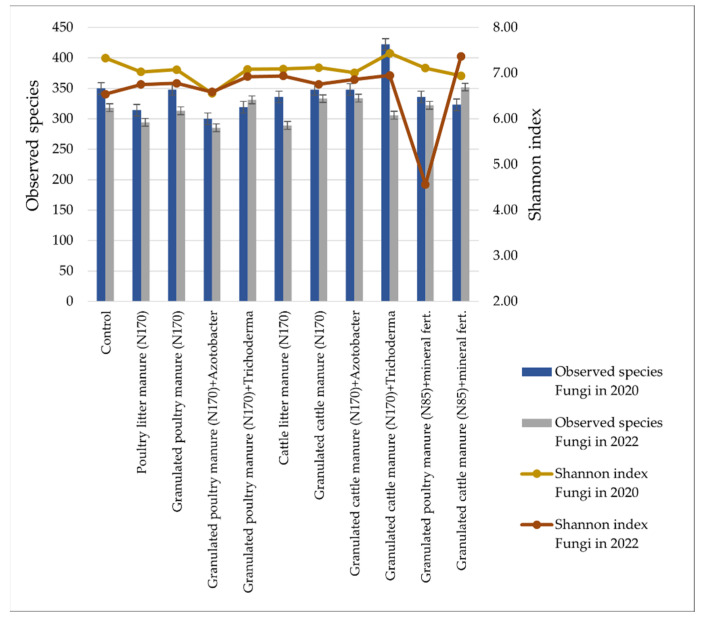
Fungal alpha diversity parameters (observed species and Shannon index) in the different fertilization options.

The following figure shows the values of fungal alpha diversity parameters in the different fertilization options (Figure 10).

The lowest diversity of fungal species was found in GPM_170_ + A soil samples (Figure 10). The highest diversity of fungal species was in GCM_170_ + T (in 2020). The Shannon index of species diversity was also the highest in GCM_170_ + T (in 2020) and lowest in GPM85 + MF (in 2022).

## 4. Discussion

This research conducted at the sites of this experiment showed that the ability of the soil to accumulate organic carbon (C_org_) is related to fertilization, both by litter and granulated fertilizers of poultry or cattle manure, and in combination with biological inputs containing *Trichoderma* spp. These trends are confirmed by studies conducted by other researchers. Fu et al. (2019) [53] showed that the application of *Trichoderma* spp. to soil increased the accumulation of organic matter and total N and P concentrations during maize cultivation. The input of *Azotobacter* spp. in soil also has a positive relationship and correlates with many soils physicochemical (e.g., organic matter, soil pH, soil moisture, etc.) and microbiological properties [54,55]. The use of *Azotobacter* spp. as a biofertilizer facilitates the release of certain nutrients, such as carbon, nitrogen, sulfur, and phosphorus, contained in soil organic matter [56]. Other researchers, after conducting experiments, also determined the positive influence of various granulated manures on soil fertility, which is directly related to the accumulation of organic matter, C_org_, humus, and mobile phosphorus and potassium in the arable layer of soil [26,57,58]. Magagula et al., 2010 [26] state that the amount of organic matter in the soil increased by 45% due to the influence of poultry manure alone, and this fertilizer also had a positive effect on other agrochemical properties of the soil. Research conducted by Raksarikorn et al., 2024 [59], showed similar trends, using both granulated organic fertilizers and mixing them with natural hormones of organic origin for plant fertilization (Cassava). Higher amounts of humus and C_org_ in the soil in autumn after white cabbage harvest were also determined by [60], using granular poultry manure for plant fertilization.

The direction and intensity of C_org_ accumulation were also determined by the granulometric composition (texture) of soil and the soil formation and microbiological processes taking place in the upper humic horizon of soil. The experimental sites belonged to the soil zone of Southeast Lithuania, which is has a light granulometric composition (texture) and less C_org_-rich soils. However, in our studies, as we can see, before the installation of the experiment, the sandy loam Haplic Luvisol, which has a subsoil of sand on gravel, mostly had medium concentrations of C_org_ and only a few plots had low concentrations. At the end of the research, a low concentration of C_org_ in the soil was found only in the control unfertilized fields, and in the fertilized fields it remained at a medium concentration, but due to the positive influence of organic fertilizers, the range of values in the soil increased. According to the scientific literature, the lighter granulometric composition (texture) of soils often leads to a slower accumulation of C_org_ in soils with lower organic matter accumulation due to poorer plant growth conditions and the infrequent rotation of perennial grasses. In addition, lighter soils heat up more easily; in these soils, due to the higher activity of aerobic microorganisms, organic matter is rapidly decomposed, and the loosened mobile mineral nutrients of plants are easily washed into the deeper layers of the soil [61]. According to other researchers, lower amounts of C_org_ are accumulated in cultivated crop rotation field soils due to more intensive mineralization processes and due to too little plant biomass formation than in the upper soil layer of meadows, and the accumulation process itself takes a significantly longer time [62,63].

In soil, mineral nitrogen (N_min_) is an indicator that characterizes the existing amount of mobile and easily assimilated nitrogen, which makes up only 1–5% of the total soil nitrogen [64]. The amount of mineral nitrogen in the soil varies significantly during the year, depending on the fertilization of plants with organic and mineral fertilizers, climatic conditions, soil genesis and granulometric composition (texture) and soil nitrogen content, and the type of cultivated plants [65]. Our research showed that the changes in N_min_ concentrations were also influenced by several of the previously mentioned factors. Very low and low N_min_ concentrations prevailed in the 0–60 cm soil layer of the experimental sites in spring. This was influenced by the prevailing meteorological conditions during the late autumn and winter periods (November–March) in the respective years. Thus, in the absence of frost, N_min_ could easily migrate from the upper soil layers of a lighter granulometric composition (texture) to the deeper ones, and then to the groundwater. In Sweden, nitrogen leaching from light-texture soils is also increased by warm winters and abundant precipitation [66]. This is also confirmed by Žičkienė et al., 2016 [67]. More intensive N_min_ leaching processes in the soil of our experiment were also accelerated by its light granulometric composition (texture), especially due to the prevailing sand on gravel in the subsoil. Rutkowska and Fotyma (2011) also confirm that the amount of N_min_ in soil is highly dependent on the granulometric composition (texture) of the soil [68]. It is known that soils with a heavier granulometric composition (texture), containing more clay and dust particles, accumulate more N_min_ stocks than those with a light granulometric composition (texture), from which nitrogen migration occurs most intensively [69,70]. According to various authors, fertilization using composted or simple manure does not allow the active leaching of nitrogen from soil, and especially from sands and loams [71,72,73].

Fertilization with various organic fertilizers also affected the changes in N_min_ in soil. Slightly more of this mobile nutrient was found in soil when plants were fertilized with both litter and granulated poultry manure, especially in combination with a biological input containing nitrogen-fixing bacteria *Azotobacter* spp. Granulated cattle manure used for plant fertilization in combination with *Azotobacter* spp. was also effective. Other researchers have also confirmed that *Azotobacter* spp. increase the amount of nitrogen in the soil by using carbon for nitrogen fixation [74,75], and whose role is very important in maintaining soil fertility and organic and sustainable crop production [75,76]. In addition, *Azotobacter* spp. can convert nitrogen into ammonia, which is then absorbed by plants to ensure the optimal process of their nutrition [77]. *Azotobacter* spp. facilitate the release of certain nutrients, such as nitrogen, sulfur, and phosphorus, present in soil organic matter [56,78]. In our experiment, the use of *Trichoderma* spp. was less stable and the effectiveness of N_min_ only appeared at the end of the trials. According to Halim et al., 2023 [79], *Trichoderma* spp. are biofertilizers that fix nitrogen, solubilize soil phosphates, and promote plant growth, as well as improve soil health and fertility. According to researchers from other countries, the biological input, *Trichoderma* spp., as an organic product, can promote the dissolution and accumulation of macro- and micronutrients that are important in plant nutrition from soil organic matter. In addition, *Trichoderma* spp. not only increase the availability and efficiency of nutrient content, but also have a positive effect on root biomass formation [53,80,81]. Based on the research conducted by other researchers, it was found that, when growing white cabbage with granular poultry manure fertilizers, the amount of total nitrogen in the soil remained higher, but the amount of N_min_ was lower, which further reduced the possibility of excess nitrogen leaching from the soil [60]. Stepantsova et al., 2021, state that increasing the rates of granulated organic fertilizers contributed to the increase in easily hydrolyzable nitrogen and other major nutrients and trace elements in the soil [82]. According to Schlegel and co-authors, the abundant content of organic matter in organic fertilizers not only improved the chemical, physical, and microbiological properties of soil, but also increased its productivity [83]. Using poultry manure maintained a stable amount of nutrients in the soil, including nitrogen, and reduced the possibility of leaching of mineral fertilizers [84].

Soil reaction (pH_KCl_) is closely related to the specific area where soils are formed, so this indicator is greatly influenced by the geomorphological situation of that area, soil-forming processes occurring in the soil, its granulometric composition (texture), species composition of growing plants, moisture regime, soil biological activity, and fertilization [85,86,87]. In our experiment, the moderately acidic reaction of soils was caused not only by the prevailing *Haplic Luvisol* in the area, which is naturally more acidic due to the intensity of substances (including calcium and magnesium carbonates) leached from the soil with precipitation, but also by the depth of subsidence of the carbonate layer itself, which is found deeper in the area of the Eastern country (at a depth of 0.8–1.2 m) than in the Middle zone [88]. In the experiment, when evaluating the efficiency of fertilization for the mentioned indicator, trends emerged that the influence of organic fertilizers of both litter and granulated cattle manure on pH_KCl_ changes in the soil was greater, when they were evaluated during the experiment, and compared to fields fertilized with various poultry manures during the corresponding 4-year period. The influence of biological inputs both with *Trichoderma* spp. and with *Azotobacter* spp. was determined, and the change in the mentioned indicator in the soil did not occur. Thus, soil pH_KCl_ still depends more on soil typology and its granulometric composition (texture) and the depth of carbonate subsidence than on fertilization with various granulated organic fertilizers. Most researchers also state that the granulated organic fertilizers used for fertilizing plants did not significantly affect the pH changes in the soil, and it remained stable throughout the research period [26,59,60]. However, Buryak et al. (2023) state that organic granular fertilizers tended to increase not only the C_org_ concentration, but also soil pH [57]. According to other researchers, *Trichoderma* spp. can reduce soil pH and activate nutrient release in soil by releasing various organic acids [53,89]. According to Kizilkaya (2009), *Azotobacter* spp. in soil correlates with many soils’ physicochemical parameters (e.g., organic matter, soil pH, etc.) [54].

In the context of global warming, ecosystems, including soil, which are able to conserve C, are very important [90]. Soil microorganisms perform important functions in soil, and one of them is the decomposition of organic material [91,92]. During normal tillage, bacteria predominate in the soil, whereas fungi predominate no-tillage conditions. Also, the presence of bacteria and fungi in the soil depends on soil chemical composition, moisture, pH, and structure [93]. In carrying out these studies, we wanted to compare the microbiological parameters of the soil in the experimental agricultural field using different organic fertilizers. 

Our research showed that bacteria belonging to two phyla—*Actinomycetota* and *Pseudomonadota* and the fungal phylum *Ascomycota*—dominated in all studied treatments. Ge et al. [94] and Liang et al. [95] also found in their research that the dominant taxonomic group of bacteria in soil fertilized with organic fertilizers was *Pseudomonadota*. After comparing the results with the control, it was observed that *Actinomycetota* was more abundant in most treatments after the application of organic fertilizers. Zhao and co-authors also report in their research that specific groups of bacteria, including *Actinomycetota*, are enriched by organic fertilization [96]. There were only a few treatments where a decrease in *Actinomycetota* after fertilizer application was observed. After the application of cattle manure, no clear trend was identified. *Bacillota* increased almost in all treatments after fertilizer application. Other researchers have observed in their studies that representatives of *Pseudomonadota* and *Bacillota* are abundant in soils fertilized with organic fertilizers [97,98]. According to Francioli and co-authors, microbial groups, such as *Bacillota* and *Pseudomonadota*, can degrade complex organic compounds, so soils fertilized with organic fertilizers promote an increase in their abundance [99]. In GPM_170_ + A and GCM_170_ + A treatments, where the bio-input *Azotobacter* was additionally applied, metagenomic analysis did not present exceptional results showing an increase in these representatives. These embedded bacteria appeared to have “dissolved” into the soil microbial background by mid-summer, when samples were taken for NGS analysis, so they were not detected during the taxonomic assignment. Overall, both organic and inorganic fertilizers should have a positive effect on the most typical soil bacterium, *Azotobacter* [16]. According to Cinnadurai et al. and Adediran et al., fertilization with inorganic and organic fertilizers increases the numbers of *Azotobacter*, but genetic diversity remains unaffected [100,101].

*Ascomycota* was the dominant phylum in all treatments, followed by *Basidiomycota*, which are consistent with data from the literature. After comparing the results with the control, it was observed that *Ascomycota* decreased in most treatments after the application of poultry and cattle manure fertilizers. Only in a few treatments after the application of cattle manure fertilizer, an increase in the abundance of *Ascomycota* was observed. Meanwhile, representatives of *Basidiomycota* increased in all fertilization options in 2022 compared to the control. According to Semenov et al., trends in fungal abundance and diversity can be highly variable with organic fertilizer application [102]. The application of organic fertilizers can increase fungal abundance and diversity, decrease them, or they may remain unchanged [102,103,104,105,106]. Ye et al. state that fertilizer-induced decreases in fungal diversity occurred most in soils with an initial pH > 6 [105]. Meanwhile, Semenov and co-authors and Xiang and co-authors state that the fungal abundance and fungal community structure are primarily determined by the total amount of organic carbon and not by soil pH [102,106,107,108]. After comparing the results with the control, it was observed that *Mucoromycota* was more abundant in most treatments after the application of organic fertilizers. According to Xiang et al. and Sun et al., the application of manure increased the relative abundance of certain favorable fungal taxa, which suppress crop pathogens [103,106]. The metagenomic analysis showed that, in the GPM_170_ + T treatment, where the bio-input *Trichoderma* was additionally applied, more individuals in the genus level, belonging to *Trichoderma,* were identified in 2020 and 2022, compared to the treatment where the bio-input *Trichoderma* was not used. Fungi of the genera *Trichoderma* are known not only as decomposers of organic matter, but also as biological control agents against plant pathogens and opportunistic avirulent plant symbionts [102].

## 5. Conclusions

Organic fertilizers and their combinations with biological inputs or mineral fertilizers effectively enriched sandy loam *Haplic Luvisol* with organic matter, thus increasing its productivity and sustainability. The positive influence of fertilization appeared on all the studied indicators, although N_min_ concentrations also strongly depended on meteorological conditions in the autumn–winter period, and soil pH_KCl_ on the granulometric composition of the soil and the depth of carbonate subsidence. Higher concentrations of C_org_ and N_min_ in the soil were also influenced by bio-inputs used in combination with various organic fertilizers. *Trichoderma* spp. fungi promoted C_org_ more effectively (1.69% and 1.62%), while *Azotobacter* spp. bacteria promoted N_min_ (4.63 mg kg^−1^ and 4.52 mg kg^−1^) accumulation in the soil. When evaluating both litter and granulated organic fertilizers of different types, it was found that higher concentrations of C_org_ and N_min_ accumulated in the soil when plants were fertilized with granulated poultry manure, and pH_KCl_ with cattle manure.

The results of this study also show that organic fertilizers and their combinations with biological inputs or mineral fertilizers cause changes related to the relative abundance and diversity of soil bacteria and fungi. Specific groups of bacteria linked to fundamental nutrient cycling, such as *Bacillota*, were promoted by organic fertilization. However, the abundance of *Pseudomonadota* and *Ascomycota* decreased in most treatments after the application of poultry manure. The results of the metagenomic analysis confirm that the use of biological inputs increases the relative abundance of *Trichoderma* spp. fungi. A treatment that used granulated poultry manure and the biological input *Trichoderma* spp. differed in the diversity of bacterial species. In this treatment, in 2022, the highest value (9.11 ± 0.017) in the Shannon index was determined. Meanwhile, the highest diversity of fungal species (7.42 ± 0.003) was observed in 2020 in the treatment that used granular cattle manure fertilizers and the biological input *Trichoderma* spp. The poorest (4.54 ± 0.007) in terms of species diversity was the fertilization option, which used granular poultry manure fertilizers with a combination of mineral fertilizers in 2022. The use of organic fertilizers is likely to increase in the future. Therefore, in the context of climate change, the long-term studies of soil agrochemical and microbiological indicators are gaining increasing importance.

## Figures and Tables

**Table 1:** Treatments used in the experimental agricultural field.

Treatment	Description
C	control (without any fertilizer—N_0_P_0_K_0_)
PLM_170_	poultry litter manure (N_170_) ^3^
GPM_170_	granulated poultry manure (N_170_) ^3^
GPM_170_ + A	granulated poultry manure (N_170_) + bio-input No. 1 ^3^
GPM_170_ + T	granulated poultry manure (N_170_) + bio-input No. 2 ^3^
CLM_170_	cattle litter manure (N_170_) ^3^
GCM_170_	granulated cattle manure (N_170_) ^3^
GCM_170_ + A	granulated cattle manure (N_170_) + bio-input No. 1 ^3^
GCM_170_ + T	granulated cattle manure (N_170_) + bio-input No. 2 ^3^
GPM_85_ + MF	granulated poultry manure (N_85_) ^2^ + mineral fertilizers (N_60_) ^1^
GCM_85_ + MF	granulated cattle manure (N_85_) ^2^ + mineral fertilizers (N_60_) ^1^

^1^ N_60_ kg ha^−1^ nitrogen fertilizer rate. ^2^ N_85_ kg ha^−1^ organic fertilizer rate calculated based on 85 kg ha^−1^ of nitrogen-active substance. ^3^ N_170_ kg ha^−1^ organic fertilizer rate calculated based on 170 kg ha^−1^ of nitrogen-active substance.

## Data Availability

Data are contained within the article.
